# Reconstruction of 3D Pavement Texture on Handling Dropouts and Spikes Using Multiple Data Processing Methods

**DOI:** 10.3390/s19020278

**Published:** 2019-01-11

**Authors:** Niya Dong, Jorge A. Prozzi, Fujian Ni

**Affiliations:** 1 School of Transportation, Southeast University, No.2 Sipailou, Nanjing 210096, nifujian@gmail.com; 2 Cockrell School of Engineering, the University of Texas at Austin, 301 E. Dean Keeton Street, ECJ 6.112, Austin, TX 78712, USA; prozzi@mail.utexas.edu

**Keywords:** laser sensor, macro-texture, micro-texture, spike, dropout, Butterworth’s filter, moving average filter, mean profile depth

## Abstract

Tire–pavement interactions, like friction and rolling resistance, are significantly influenced by pavement macro-texture and micro-texture. Accurate texture measurement at the micro-texture level is vital to achieve the desired level of safety, comfort, and sustainability of the pavement. However, the existence of dropouts and spikes in the collected data is still inevitable based on current laser devices, which leads to erroneous texture characterization. This study utilized an advanced laser sensor to measure three-dimensional (3D) pavement texture at the micro-level at a given speed. Using a proposed interpolation method, the dropout areas in the raw measurements were filled up. Butterworth’s high-pass and low-pass filters were applied to separate two texture components from the profile. Based on a statistical analysis for the micro-texture amplitude, an appropriate threshold was determined in order to identify the spikes. A three-step-spike-removal method was proposed and found to be effective in clearing the spikes. The 3D pavement profiles were finally reconstructed without dropouts and spikes. Mean profile depth (MPD) was calculated with different baselines. It was found that the presence of spikes leads to a greater MPD value and the MPD is sensitive to the baseline length. A shorter baseline is recommended to mitigate the impact of spikes on the accuracy of the MPD.

## 1. Introduction

The tire–pavement interaction is essential to the safety of motorists. The direct force generated in the tire–pavement interface is known as skid resistance, which is defined by the properties of the pavement surface texture, the tire, the vehicle speed and the environmental condition. Pavement surface texture plays an important role in road friction, tire–pavement noise, splash and spray, and rolling resistance. Adequate surface texture of a pavement is essential to providing good skid resistance, preventing accidents, reducing splash and spray, and mitigating tire–pavement noise among other benefits [1,2]. The pavement surface texture can be influenced by many factors, including mix gradation, aggregate size and type, and texture orientation, among others. 

Pavement texture is defined as the deviation of the surface layer from a true planar surface [3]. The Permanent International Association of Road Congresses (PIARC) has categorized surface textures based on the wavelengths and amplitudes of the deviations. The standard specifications, such as the American Society of Testing Materials (ASTM) [4], and German Institute for Standardization (DIN on ISO 13473-1) [5], have accepted and incorporated these definitions. According to the ISO 13473-1, the pavement texture is generally classified into four categories:

- Unevenness (500 mm < wavelength < 50 m)
- Mega-texture (50 mm < wavelength < 500 mm, 0.1 mm < amplitude < 50 mm)
- Macro-texture (0.5 mm < wavelength < 50 mm, 0.1 mm < amplitude < 20 mm).
- Micro-texture (wavelength < 0.5 mm, 1μm < amplitude < 500 μm).

These texture scales have different influences on tire–pavement friction. Unevenness is usually caused by permanent deformation due to traffic loading or construction defects. It leads to poor ride quality and bad pavement drainage as well as reduced tire-pavement contact [6]. The mega-texture is described by potholes, pavement rutting, cracks and major joints. It affects vibrations in the tire walls and is therefore highly associated with rolling resistance and noise. The macro-texture is generally formed by the size and shape of the aggregate particles in the pavement surface. It is responsible for a large portion of tire–pavement friction at high speeds on wet pavements [7]. The micro-texture is the surface roughness of a pavement at the microscopic level [8]. It is typically described as the fine-scale texture existing on the surface of the aggregate particles in the pavement surface. It has a close interaction with the tire rubber and plays a significant role in skid resistance on both wet and dry pavements [6]. For optimum pavement performance, both micro-texture and macro-texture are essential, while mega-texture and unevenness are undesirable [9]. 

Traditional methods for measuring the pavement surface texture are the sand patch, the grease patch, and the outflow meter. However, these methods are inefficient as they can only measure the surface depth at a discrete spot on the pavement, and their measurements cannot shed any light on the micro-texture. Modern methods that use laser or optical sensors are able to scan the surface texture and calculate the indicator, such as mean profile depth (MPD). Some of these methods are only capable of providing a spot measurement, such as the circular track meter (CTM), while others can measure the texture continuously along the pavement at high speeds, such as the laser cracking measuring system. Currently, none of the commonly used devices for pavement texture scanning can capture the micro-texture component at high speeds due to the limitations of the resolution and sampling rate of their sensors [10].

Due to the continuous progress made in electronics and sensors in recent years, sensor devices with higher resolution and sampling rate are now available. Meanwhile, close range photogrammetry in industry has become more and more popular and successful with rapid development in automated and highly accurate 3D measurements [11]. These devices have been used in a variety of applications in many fields, and allow the acquisition of objects on very fine scales and in a wide range of sizes [12]. For the photogrammetry systems utilizing high-resolution digital cameras with wide angle lenses, sub-pixel image point measurement operators provide object measurement within minutes using robust bundle adjustment, including self-calibration [13,14]. The precision of the image point measurement can be as high as 1/50 of a pixel, yielding typical measurement precision in the range of 1:100,000 to 1:200,000, the former corresponding to 0.1 mm for an object of 10 m size [15,16]. The absolute accuracy of length measurements is generally 2–3 times less (i.e., about 0.05 mm for a 2 m object) than the precision of object point coordinates, which illustrates the relative accuracy of 3D shape reconstructions [17]. The successful use of photogrammetry in industry requires a number of technical components including sensor resolution, verification of accuracy, data acquisition, data compression, data processing, methods for determination of 3D reconstruction coordinates and error statistics and so forth. In summary, appropriate design, setup and operation of close-range photogrammetry systems forms a complex task. The feasibility of a solution is not only a technical question but also a function of required documentation, system support, cost–performance ratio, quality assurance and interdisciplinary skills [11].

State-of-the-practice methods commonly used for measuring pavement texture only focus on the macro-texture, which alone may not be sufficient for the effective evaluation of skid resistance. Therefore, in order to obtain both macro-texture and micro-texture components of the pavement surface, more advanced sensor devices, which can measure the roughness at a microscopic level are needed to be employed for pavement texture investigation. 

According to practice experiences, the raw measurements obtained from such advanced sensor devices, still have some defects such as the presence of noise in the form of dropouts and spikes. A National Cooperative Highway Research Program (NCHRP) report indicated that the analysis of profile data collected by Long-Term Pavement Performance (LTPP) profilers revealed problems due to spikes in the data [18]. A study conducted for the Federal Highway Administration (FHWA) also revealed problems with spikes in the data taken for international roughness index measurements [19]. Other researchers [20,21] also observed the presence of spikes in pavement profile measurements and suggested that the change in surface reflectivity caused by water, silica, pavement markings, polished aggregates etc., could potentially be the source of these spikes. In addition, there are many factors that can lead to the presence of noise in the measurements. For example, shiny mirror-like surfaces change the amount and direction of light reflected to the receiving lens, and black materials scatter only a small part of the incident light. Black and shiny materials (like fresh asphalt) have both problems simultaneously. Transparent and semitransparent materials (pavements with such minerals in the aggregate, reflective paint on the road etc.) will cause offsets in the reflection and will absorb or deviate a portion of the light [22]. Other factors, such as temperature, geometry, secondary reflections, bandwidth, and sampling rate, are potential sources of noise in the measurements.

The presence of dropouts and spikes will result in inaccurate measurements and erroneously calculated texture indicators, which may also hinder the adoption of the advanced laser devices. Therefore, in order to accurately measure the pavement texture, there is a pressing need for methods to deal with dropouts and spikes. A previous study [23] stated that besides the slope suppression, drop out correction and traditional low-pass filtering, the calculation of the MPD must be free of erroneous data like spikes. Katicha et al. [24,25] proposed methods for identifying and removing spikes by controlling the false discovery rate or wavelet transform analysis, which were confirmed to be effective for pavement macro-texture measurements. 

It could be said that the presence of spikes is seemingly inevitable in raw collected measurements, though there have been methods proposed to deal with this problem. However, it is important to note that due to the totally different laser devices used for investigations, the spikes in terms of the amplitude, the frequency and the wavelength are not necessarily the same in different studies. Furthermore, previous methods for spike removal only involved the macro-texture component of the pavement texture, while in this study both the macro-texture and the micro-texture were involved. Consequently, the previous methods are not necessarily applicable in any situation. Based on these facts, new methods are needed to be developed in order to handle dropouts and spikes for this study, where a more advanced laser device was first introduced to scan the pavement texture.

The main objectives of this study were to:

1. Propose a method that can easily fill up the dropout area in the pavement profile.
2. Develop a method that can identify and remove the spikes from the pavement texture measurements.
3. Validate the above-mentioned methods by construction of a 3D surface profile and calculation of the mean profile depth (MPD) based on the original and corrected measurements.

**Figure 1:**
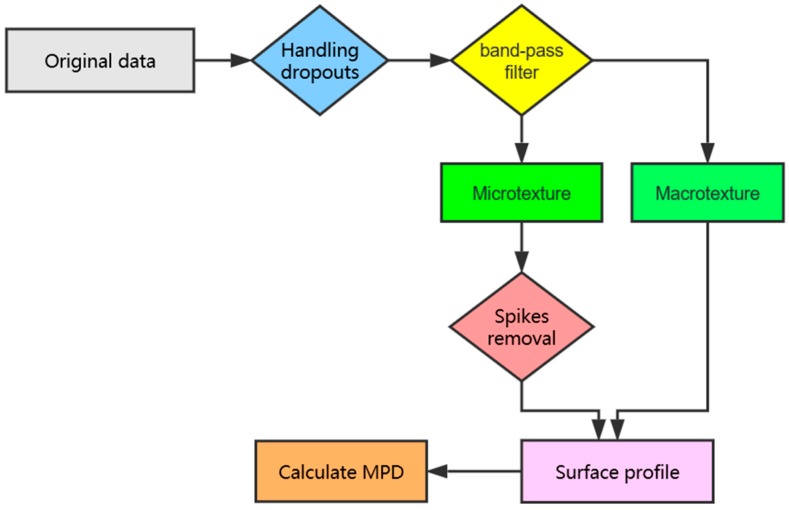
Flow chart of implementation procedures.

Figure 1 shows the technological flow of data processing in this study. In this flowchart, the position where a rhombus appears indicates that a specific technique is applied, and where a rectangle appears, a batch of data is generated. In summary, there are three main steps to be taken including handling dropouts, applying a high-pass/low-pass filter, and removing spikes. Finally, the corrected pavement profile consisting of both macro-texture and micro-texture was obtained for the reconstruction of a 3D pavement texture and calculation of the MPD value.

## 2. Measurement Method

### 2.1. Test Device

**Figure 2:**
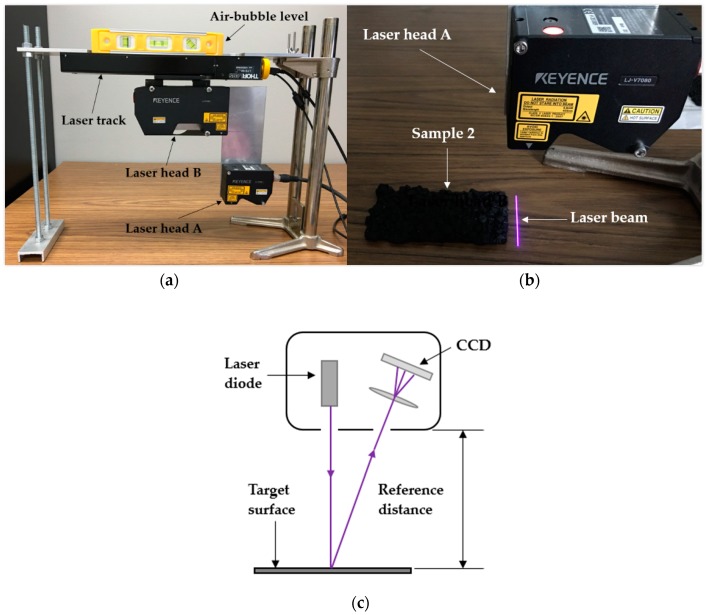
(**a**) The prototype including the frame and mounted lasers; (**b**) scan application with laser head A; (**c**) the triangulation system of a laser [6].

In this study, all parts of the laser equipment used were made by Keyence in Japan. As illustrated in Figure 2a, this laser equipment involves a LJ-V7001P controller (with a linear track) and a CV-U3 power source, with two different laser heads mounted onto the supported metal plate. The bigger one was fixed on the linear track by which both of the lasers can be driven to perform uniform motion at a given speed from the right to the left. An air-bubble level was put on top of the system to ensure the correct horizontal condition of the test environment. Each laser has a specific scan range and resolution. The laser A (Model number: LJ-V7080) with different scan accuracies along two horizontal directions (0.005 mm along the motion direction and 0.05 mm along the laser light direction) was chosen to carry out the scan work due to its higher resolution than laser B (Model number: LJ-V7300). 

When the system is turned on, as shown in Figure 2b, a purple laser beam in a horizontal line is projected from an emitter in the laser head and then the reflection of the light from the sample surface is captured by a detector. If the angles between the projected light and the reflection are known, then by using a triangulation method, the system can calculate the height profile of the surface relative to the system’s reference line. Small changes in the height due to texture irregularities can also be captured using this system [26]. Figure 2c illustrates the schematic drawing of the triangulation system [6]. The system may have missed some data from the vertical edges as a result of the angle between the laser and the camera.

When static, this device is only capable of scanning two-dimensional (2D) depth data from a given surface. For the purpose of measuring three-dimensional (3D) surface textures, the laser head was mounted onto a motion controller so as to travel over the sample surface and scan the object at a given interval (this interval can be changed to accommodate the desired sampling rate). According to the device manual, a vertical reference distance of approximately 80 mm should be kept between the laser head and the object to be scanned in order to achieve the maximum scan width of 48 mm. Therefore, the width of the sample for scanning should be within 48 mm, otherwise the area beyond the coverage of the laser line cannot be scanned and measured by the system. In addition, both the laser head and the motion controller were connected to a computer. The software LJ-Navigator was used to monitor the measurement process and acquire the data. Within this software, the measurement range, frequency of sampling, the motion speed, acceleration etc. are adjustable as needed, according to the target. 

### 2.2. Test Settings

The batch mode in the software LJ-Navigator was used to record measurements during scanning. In this mode, a given number of points can be recorded during an entire single scan. Each point includes a specific height value for the surface texture. The greater the number of points selected, the less the resulting sampling interval. The settings for the pavement texture scan in this study are noted in Table 1. For each scan, the recorded number of points per batch were selected as 15,000, which is the maximum number available to collect in this mode. The moving velocity of the laser head was set at 5 mm/s. Thus, the corresponding sampling frequency was 1 kHz along its moving direction, indicating that 1000 points can be recorded over one second with a sampling interval of 5 μm (0.005 mm). Based on these settings, the effective scan distance that the laser system could measure was 75 mm. In addition, it is worth noting that the sampling interval along the laser beam line is not adjustable and was fixed at 0.05 mm by the system. As a result, the collected data for each scan was in the form of a 15,000 × 800 matrix, which can be exported in a simple CSV data file.

### 2.3. Sample for Measurement

**Figure 3:**
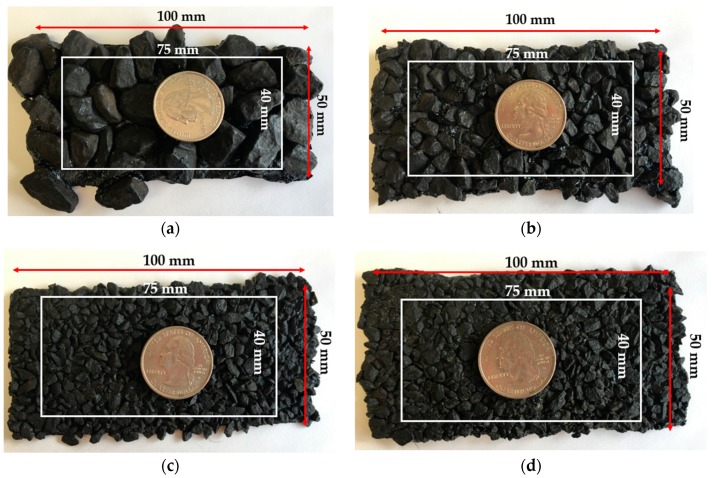
Pavement samples used for scan, (**a–d**) for samples 1–4.

As illustrated in Figure 3, four rectangular pavement samples with different gradations were evaluated in this study. The samples have approximately the same dimensions—100 mm long and 50 mm wide. Due to different sampling intervals in two horizontal directions, as marked by a white rectangle in the figure, the effective maximum measured area for each pavement sample was as long as 75 mm and as wide as 40 mm. 

## 3. Handling Dropout

A dropout is a data point with a meaningless value designated by the laser system. In other words, the value is apparently erroneous and cannot represent the true height of the position. In this study, all of the dropouts were designated to be −99.99 mm by the laser system, so it was easy to find the dropout areas. Before correcting these dropouts, it was necessary to understand the scope and extent of them.

### 3.1. Dropout Distribution

**Figure 4:**
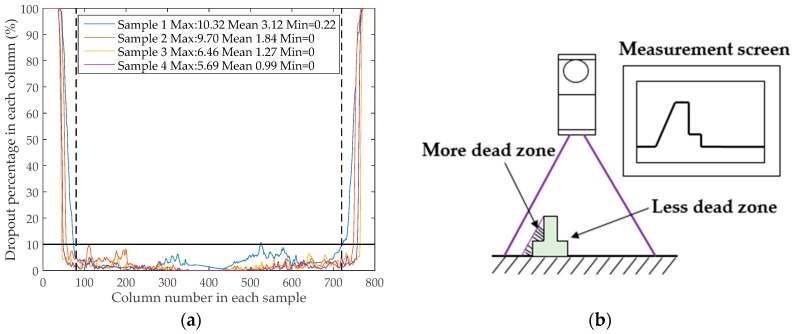
(**a**) Distribution of the dropout percentage in different areas for each sample; (**b**) the formation of the dead zone during scanning [26].

As mentioned earlier, the collected data was in the form of a 15,000 × 800 (row/length × column/width) matrix. A such, the 3D surface texture for each sample was comprised of 800 lines with each line including 15,000 points. Figure 4a shows the distribution of the dropout percentage of each line/column for each sample. It is apparent that the distributions of the dropout percentage among these samples are similar. More specifically, the dropout percentage grows sharply to 100 as the column number becomes close to either 0 or 800, indicating that the dropout area becomes larger as the distance from the edges decreases. By contrast, the dropout percentages in the middle (column number around 400) of the sample are moderate, with the amplitude fluctuating between 0 and 10. 

In light of these facts, it is suggested that the area with the lower dropout percentage could be filled up by interpolation according to the effective reference values around it. However, as the dropout percentage becomes extremely high, the reference values hardly exist. Thus, it is impossible to calculate reasonable values for the dropout area. For this reason, the columns/lines with an extremely high dropout percentage should be dismissed. Finally, the data between lines 81 and 720, which are marked between the dashed lines in Figure 4a and have a dropout percentage no more than 10, were selected for use in the subsequent procedures. 

As per Figure 4b, a dead zone here refers to an area within the scan coverage of the laser line which cannot be identified and measured correctly. The existence of dead zones during scanning process directly leads to the occurrence of dropouts in the collected data. 

The formation of dead zones or dropouts can be explained as follows: On one hand, it can be thought that when the object is located right under the emitter (in the center of the laser line), the laser light projected from the emitter has the minimum incident angle. When the object moves along the laser line from the center to the left or right side, the incident angle increases. The growth of incident angle will certainly lead to the same amount of growth in the reflex angle. When the incident angle reaches to a certain threshold where the corresponding reflex angle cannot be received by the detector in the laser head, dropouts are thus produced. In other words, due to the locations out of the effective coverage of the laser line, dropouts occur in the measurements. This could explain why the dropout percentage increases sharply to 100 adjacent to the two edges of the sample, as shown in Figure 4a. 

On the other hand, although the object is located within the effective scan coverage, dropouts can still be present because the object for scanning is not smooth, especially for the pavement texture in which the aggregates create too many peaks and valleys. As Figure 4b illustrates, the peaks can partly or completely block the irradiation of the incident light, which creates the dead zones and then results in the dropouts in the measurements. This could be the reason why the dropout percentage is relatively more significant from coarser samples, as shown in the box in Figure 4a.

### 3.2. Rectification Method

The column/line with the maximum dropout percent of 10.32 for Sample 1, was selected as the representative in the following processing methods. 

**Figure 5:**
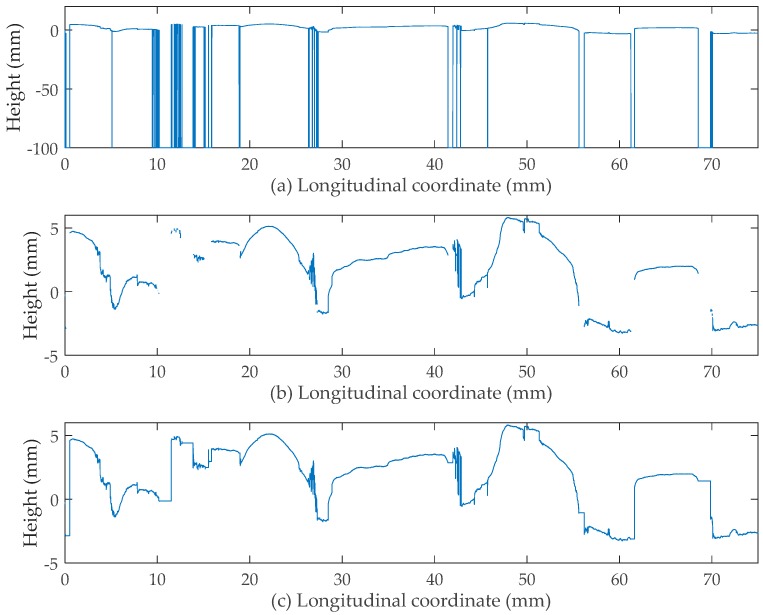
(**a**) Original measurements; (**b**) measurements with dropout value dismissed; (**c**) measurements with dropout corrected.

The literature on handling dropouts is undeveloped and thus, a method to address this problem is proposed here. As can be seen in Figure 5b, due to the presence of dropouts, the original output profile is discrete. With the aim to correct these discontinuous parts, the procedure developed an algorithm to predict appropriate values assigned to the dropout points. Firstly, for the dropout point from number 6 to 15,000, the measurements from five non-dropout points ahead of it were averaged and determined as the prediction. Secondly, for dropout from number 1 to 5, the mean value of its latter five non-dropout measurements was calculated as the predicted value.

For the increasing order of the i-th point from 6 to 15,000, Equation (1) was applied where point i was judged as a dropout. With the above procedure done, Equation (2) was then applied to the i-th point from 5 to 1. Note that these two steps cannot be exchanged so as to achieve the optimum output.

(1) $$h_{i}=1/5\times \overset{5}{\underset{j=1}{\sum }}h_{i-j}\;(i\geq 6)$$

(2) $$h_{i}=1/5\times \overset{5}{\underset{j=1}{\sum }}h_{i+j}\;(1\leq i\leq 5)$$

where

i, j = longitudinal position of point i or j, and
$h_{i}$ = measurement of point i, and
$h_{i\pm j}$ = measurement of point i±j.

### 3.3. Comparison before and after Correction

As shown in Figure 5a, the vertical line represents the presence of a dropout where the measurement was designated as −99.99 mm by the laser system and the blank stripe indicates a large number of dropouts are continuously present. Figure 5b shows the discrete profile with all dropout values wiped off. The corrected profile can be seen in Figure 5c. It is clear that the discontinuous parts are all connected together, proving the effectiveness of the method. However, the corrected profile still has some defects that cannot perfectly represent the true profile, such as the presence of the right angles. Because the proposed algorithm only used the measurements either ahead of or behind the dropout, a right angle was created where a large number of dropouts existed previously. Improvements need to be made in future studies in order to make the corrected profile more accurate. Although imperfect, the method still enjoys simplicity and efficiency in its operation.

## 4. Spike Removal

### 4.1. Theoretical Background for Pavement Profile Analysis

Pavement profiles can be treated as signals of a physical variable with which a datum is associated. The physical variable is represented by texture elevation, while the independent variable is the spatial distance. Generally, signals are defined in a time domain, although the independent variable may not be time, as is the case to be examined here. 

The pavement surface profile is a continuous signal that is recorded with a discrete sampling interval where the amplitude is ∆x. A y(x) sequence is thus obtained, which can continuously take on all the real values contained in a given range. In the profiles recorded, the trend of the profile cannot be generally known a priori but rather only after being measured.

Given these features, pavement profiles can be defined as discrete-time, continuous amplitude, aperiodic, and random signals. The unlimited set of all the profiles that can be surveyed on a pavement is a random process. For discrete-time random signals, the theory of signals defines an important property—stationariness. Pavement profiles meet the requirement of stationariness because if pavement is considered with homogeneous surface characteristics, then all the profiles measured are equivalent from a statistical point of view.

The analysis of random signals is carried out by means of filters. For example, mono-dimensional systems, with which a transformation can be performed so that an input signal x(t) will be matched by a well-determined and single output signal y(t).

### 4.2. Application of Butterworth’s Filter

The Butterworth filter is a type of signal processing filter designed to have a frequency response which is as flat as possible in the passband. It is also referred to as a maximally flat magnitude filter. It was first proposed in 1930 by the British engineer and physicist Stephen Butterworth in his paper entitled “On the Theory of Filter Amplifiers” [27]. In this study, Butterworth’s numerical high-pass and low-pass filters were used to separate different texture components. There are several reasons for choosing it. Firstly, it has been successfully implemented in many commercial tools, such as Matlab (Matlab 2015b was used in this study), making it easily accessible to users. Secondly, it provides a maximally flat amplitude response |H(f)| in the pass band, which means that for the n-order filter, all derivatives of ${\left|H(f)\right|}^{2}$ up to but not including the 2n-th derivative are zero at f = 0. In addition, it has a monotone amplitude response both in the pass band and in the stop band. As shown in Equations (3) and (4), the amplitude responses |H(f)| of a Butterworth low-pass filter $\left|{H(f)}_{\mathrm{LP}}\right|$ and high-pass filter $\left|{H(f)}_{\mathrm{HP}}\right|$ have the following forms, respectively:

(3) $$\left|{H(f)}_{\mathrm{LP}}\right|=\frac{1}{\sqrt{{1+(f/f}_{c}{)}^{2n}}}$$

(4) $$\left|{H(f)}_{\mathrm{HP}}\right|=\frac{{{(f/f}_{c})}^{n}}{\sqrt{{1+(f/f}_{c}{)}^{2n}}}$$

where $f_{c}{}^{2}$ is the cut-off frequency and n is the filter order. The ${f/f}_{c}$ ratio is the normalized frequency ω. With an increasing filter order n, the filter amplitude response becomes progressively steeper; the pass band section with |H(f)|≅1 increases and the flanks of the transition region tend ever more rapidly toward 0, while the value of the cut-off frequency $f_{c}$ remains unchanged.

The sampling frequency of the laser device used was 1 kHz, with a corresponding sampling interval of 0.005 mm. Therefore, the sampling interval of 0.5 mm corresponds to the sampling frequency of 10 Hz. Because the threshold between macro-texture and micro-texture is 0.5 mm (wavelength of the texture), the cut-off frequency between them should be 10 Hz. 

**Figure 6:**
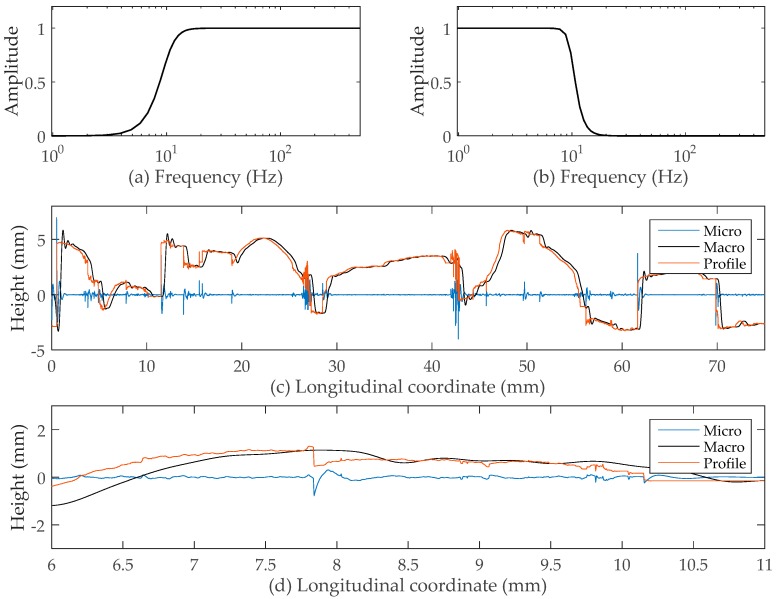
(**a**) Amplitude response of Butterworth’s high-pass filter; (**b**) low-pass filter; (**c**) profile with filtered micro-texture and macro-texture; (**d**) close-up of the profile.

Figure 6a,b shows the amplitude response of the Butterworth’s numerical high-pass and low-pass filters with a cut-off frequency of 10 Hz, respectively. In addition, the filter order (n) used in this case was set to 4. As illustrated in Figure 6c, the micro-texture represented in the blue line, was separated from the profile by the high-pass filter. Similarly, through the low-pass filter, the macro-texture was obtained, which is represented by the black line. The profile before filtering is marked by the red line. Figure 6d is a close-up view of a selected area with the longitudinal coordinate from 6 mm to 11 mm.

As can be seen, due to the absence of both spikes and micro-texture, the macro-texture that is much smoother than the unfiltered texture retains a reasonable outline of the texture. Because the wavelengths of the spikes and micro-texture were overlapping, the spikes were still kept in the filtered micro-texture. In order to obtain a clean and reliable micro-texture, more steps should be taken to identify and remove the spikes. 

### 4.3. Statistical Analysis for Micro-texture

Spikes were included into the micro-texture component by the high-pass filter, indicating that spikes were barely distinguishable from the micro-texture in the frequency domain. According to the ISO 13473-1 [5], the amplitude for the micro-texture is specified as no more than 0.5 mm. This could be a reasonable criterion to identify spikes. In order to confirm the validity of this criterion, the characteristics of the amplitude distribution for the micro-texture including spikes should be taken into consideration.

**Figure 7:**
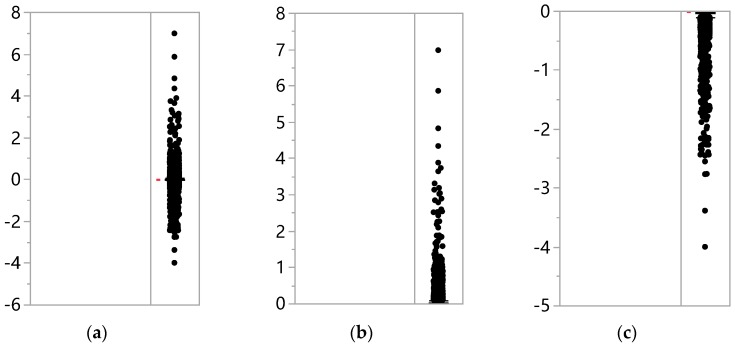
Amplitude distribution of the (**a**) whole micro-texture; (**b**) positive micro-texture; (**c**) negative micro-texture.

Figure 7 shows the amplitude distribution of separated micro-texture measurements from a statistical point of view. Figure 7a illustrates the distribution of the whole micro-texture, Figure 7b the distribution of the positive micro-texture, and Figure 7c the distribution of the negative micro-texture. 

Table 2 summarizes some important statistical indicators calculated from the above distributions. As indicated in the table, the amplitudes range from −4 mm to 7 mm; the mean and the median values for both positive and negative micro-textures were very close; 95% of the micro-texture measurements are within −0.34 mm to 0.35 mm, corresponding to less than 5% of the total proportion for spikes and hence, it is reasonable to use −0.5 mm and 0.5 mm as the thresholds to identify spikes. 

### 4.4. Application of Moving Average Filter

To remove the spikes, other methods are introduced here. The moving average filter is differentiated from other filters in its simplicity of application, which makes it the most commonly used filter in digital signal processing [3]. Furthermore, it is regarded as an excellent smoothing filter for time domain operations, especially for its ability to eliminate any noise from the signal. The transformation used in this study, which embodies the algorithm of the moving average low-pass filter, has the following relationship:

(5) $$y_{i}=\frac{1}{(B/(\Delta x+1))}\overset{i+(B/2\Delta x)}{\underset{j=i-(B/2\Delta x)}{\sum }}x_{j}$$

where

$y_{i}$ is the output value at the i-th point for this filter, and
Δx is the sampling interval or distance between points, and
$x_{j}$ is the input value, and
B is the computational base of the filter which identifies the extension of the $x_{j}$ interval which is averaged.

Unlike the Butterworth filter used earlier, the moving average filter applied in this study is exclusively targeted towards spikes rather than all points within the profile. Namely, this filter is required to only process the amplitude of the spikes without changing the frequency spectra of others. It was found that when the range of B (computational base) is too short, the corresponding filtering effect is insignificant for the area where spikes appear densely. However, when B is considerably long, the achieved filtering effect is undesirable. Based on multiple trials, it was found that the expected filtering effect can be achieved when the moving average filters are consecutively applied with B values of 15 mm, 5 mm, 1 mm, respectively.

**Figure 8:**
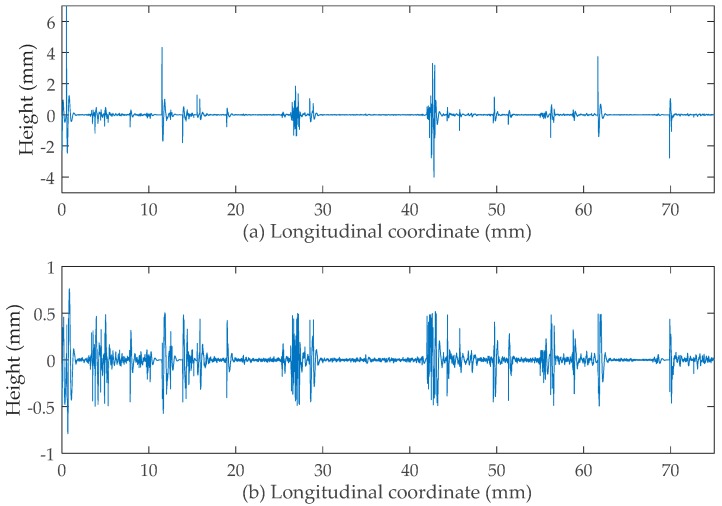
(**a**) Micro-texture with spikes and (**b**) without spikes.

Figure 8a,b shows the micro-texture profiles before and after filtering, respectively. As can be seen in Figure 8a, the amplitude range of the micro-texture is between −5 mm and 5 mm. By contrast, the amplitudes of the spikes after filtering are reduced to an appropriate value range from −0.5 mm to 0.5 mm. Consequently, this fact confirms the validity of the proposed spike removal procedures.

**Figure 9:**
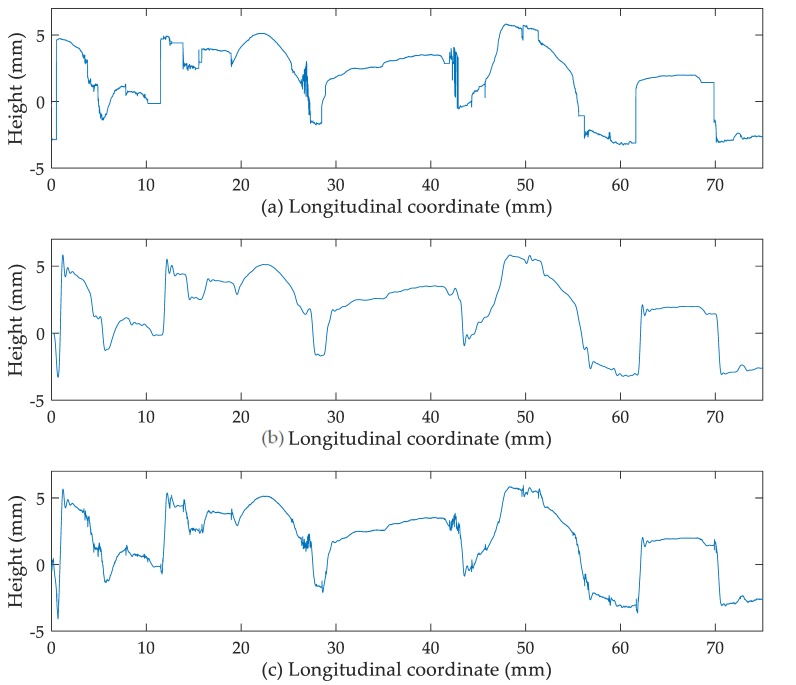
(**a**) Unfiltered profile with dropouts corrected; (**b**) filtered profile only with macro-texture; (**c**) filtered profile (macro-texture micro-texture combined together) without spikes.

Figure 9 shows a comparison between the unfiltered profile with dropouts corrected, the filtered profile only with macro-texture, and the filtered profile (macro-texture and micro-texture combined together) without spikes. It was noticed that: (1) due to the existence of spikes, the first one displays the surface texture in a distorted way; (2) only with macro-texture, the profile just displays the rough outline of the texture without details; (3) the third one best represents both the macro-texture and the micro-texture of the profile. 

From the above, appropriate results are achieved with the above methods, thus, the correction procedures can be extended and applied to the whole of the collected data so as to reconstruct the 3D surface profile without dropouts and spikes.

### 4.5. Reconstruction of a 3D Pavement Surface Profile

**Figure 10:**
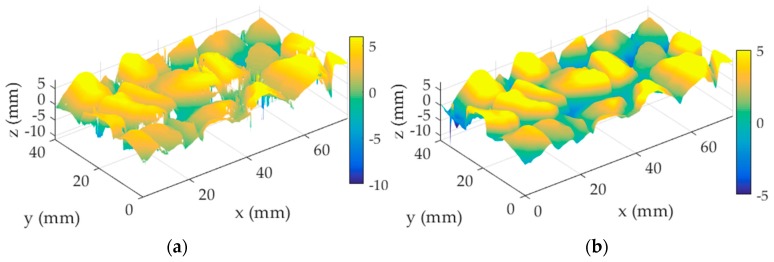

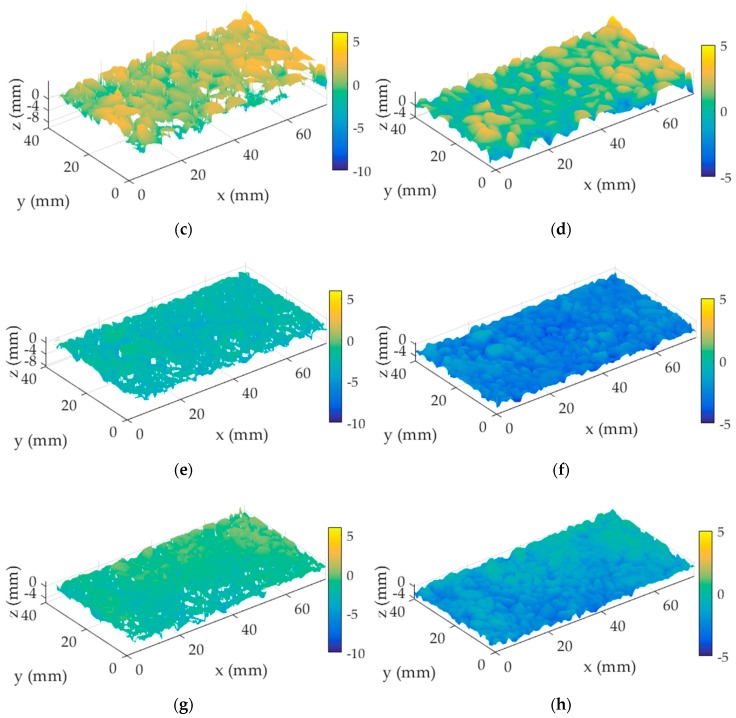
(**a–g**) Original models for samples 1–4; (**b–h**) their reconstruction models.

With the aim to obtain an accurate 3D surface texture, each sample involving 640 lines was applied to the processing techniques described above. 3D reconstructed surface models for these samples were developed. Both the reconstructed and original profiles are displayed in Figure 10. As can be seen, in the original profiles the “holes” represent the existence of dropouts and the “needles” represent the presence of spikes. By contrast, in the reconstructed profiles the “holes” and the “needles” are rarely seen. The comparison of the results visually confirmed that the qualities of 3D pavement texture constructed with corrected measurements are improved a great deal.

## 5. Calculation of MPD Value

To quantify the correction effects, the parameter of the mean profile depth (MPD) which is commonly used for characterization of pavement profile, was calculated for both the original and the corrected measurements, respectively. 

In ASTM E1845-09 [28], the calculation of the MPD is specified only for macro-texture with a baseline of 100 mm long. That is to say, the baseline length for characterization of a micro-texture has not been determined yet. As previous researchers have already done, the MPD calculation could also be applied for micro-texture with a proposed baseline length. In order to observe the influence of the baseline’s length on the MPD value, three lengths—1 mm, 5 mm, and 10 mm, were used to investigate the micro and macro texture with a short wavelength. The sampling interval was 0.005 mm, indicating that 200 measurements were collected for every one millimeter. Therefore, there are 200, 1000, and 2000 points involved for baseline lengths of 1 mm, 5 mm, and 10 mm, respectively. Each sample has 640 lines and each line includes 15,000 points (75 mm). For the baseline of 1 mm, 5 mm, and 10 mm, the resulting total number of segments is 75, 15, and 7 per line and 48,000, 9600, and 4480 per sample. According to ASTM E1845-09, the MPD value for each segment can be calculated using Equation (6):

(6) $$\mathrm{MPD}=0.5\times \left[\max\left(h_{1}{,\ldots h}_{N/2}\right)+\max\left(h_{N/2+1}{,\ldots h}_{N}\right)\right]$$

where

N = number of points within the baseline, and
$h_{i}$ = amplitude of point i.

**Figure 11:**
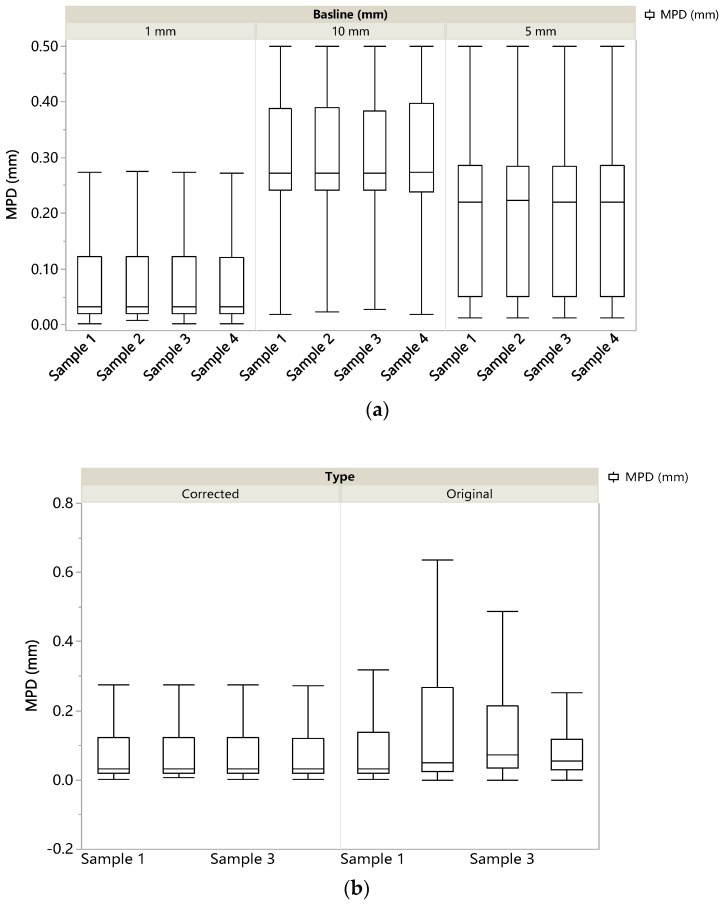
(**a**) the MPD distributions based on different baselines; (**b**) comparison of the MPD distributions between original and corrected profiles.

The MPD value for each segment was calculated. The distributions of the MPD based on corrected data with different baselines are shown in Figure 11a. It is obvious that no matter how long the length of baseline is, with the baseline increasing from 1 mm to 10 mm, the median MPD value undergoes an apparent growth. However, because the aggregate sizes among these samples are quite different, the differentiation of distribution properties of their micro-texture is insignificant. The results indicate that the indicator of the MPD for the characterization of micro-texture is insensitive to the aggregate size in this case.

Figure 11b presents the comparison of the MPD distributions (with the baseline of 1 mm) between the original and corrected measurements. It is clear that for all samples evaluated, the median MPD from the corrected data is lowered to some extent as compared to that of the original data. This proves that the presence of spikes impacts on the accuracy of the MPD.

In order to expand on the difference, the mean and median MPD values calculated with different baselines from both original and corrected profiles are listed in Table 3. 

From this table, the key findings are as follows:

1. Both the mean and the median values of the MPD undergo a significant growth as the baseline increases.
2. In general, the mean MPD is much more sensitive to spikes than the median MPD.
3. With the decrease of the baseline length, the difference of mean MPD between the corrected and original profiles becomes more and more insignificant, indicating that the errors caused by spikes in the MPD values were much reduced by choosing a shorter baseline.
4. For each sample with a given baseline length, the mean MPD value is greater than its median.
5. Compared with original results, both the mean and median MPD for the corrected profiles decreases to some extent.

From the above, it can be concluded that the impact of spikes on the MPD is sensitive to the baseline length. More specifically, when a longer baseline is used (e.g., 10 mm), the presence of the spikes will make the median MPD increase by 54 to 276 %; when choosing a much shorter baseline (1 mm), this increment is much reduced to within 118 percent. Thus, it can be inferred that when the spikes cannot be fully removed from the raw measurements, using a much shorter baseline is a good way to mitigate the impact of spikes on the accuracy of the MPD value.

## 6. Conclusions 

According to results and analyses presented above, some conclusions can be drawn as follows:

1. The occurrence of dropouts was mainly due to two reasons. On one hand, when the location of the object is out of the effective scan coverage, a large number of dropouts will appear in the data and should be dismissed. On the other hand, due to the roughness of the pavement texture, some areas may be sheltered from the laser light, which leads to small number of dropouts. It was found that the dropouts were present more frequently as the gradation of the sample became coarser. Using a linear interpolation method proposed in this study, the dropout areas in the pavement profile were filled up. Though the correction effect is not perfect, this method still confers simplicity and efficiency for operation.
2. Using Butterworth’s high-pass and low-pass filters with a cut-off frequency of 10 Hz and filter order of 4, the micro-texture and macro-texture components were separated from the measurements, respectively. However, because the spikes and micro-texture were overlapping in the frequency domain (wavelength), the spikes still remained in the micro-texture.
3. A three-step spike removal method using moving average filter was developed to remove the spikes. The spikes were identified according to the amplitude range. In order to obtain an optimum smoothing effect, the moving average filter was applied three times with different computational bases (B) from long to short. The recommended B values were 15 mm, 5 mm and 1 mm, respectively. This method was only applied to spikes without changing the frequency and amplitude of non-spike points and therefore ensured the accuracy of the micro-texture measurements.
4. The 3D pavement surface texture model was constructed to visually verify the effects of the proposed methods. It was found that the dropouts and spikes appearing in the original models no longer existed in the corrected ones, proving the effectiveness of the corrected methods proposed in this study.
5. The presence of spikes certainly caused the MPD of the micro-texture to increase to some extent. The impact of spikes on the MPD was sensitive to the baseline length. With the baseline decreasing from 10 mm to 1 mm, the MPD value saw an apparent decrease and the errors caused by spikes in MPD values were much reduced. Thus, 1 mm was recommended as a better option for characterization of the micro and macro texture with a short wavelength so as to mitigate the impact of spikes on the accuracy of the MPD.

## Figures and Tables

**Table 1:** Settings for pavement texture scan.

Numberof Points	Moving Velocity (mm/s)	Sampling Frequency (Hz)	Sampling Interval (mm)	Scan Distance (mm)
15,000	5	1000	0.005	75

**Table 2:** Statistical indicators for micro-texture with spikes.

Indicator	Micro	Micro > 0	Micro < 0
Max	6.971	6.971	$-{1.5\times 10}^{-6}$
99.5%	1.005	1.292	$-{8.26\times 10}^{-5}$
97.5%	0.345	0.645	$-{3.39\times 10}^{-4}$
90%	0.059	0.145	−0.002
75%	0.014	0.043	−0.005
Median	${2.44\times 10}^{-4}$	0.014	−0.014
a25%	−0.013	0.005	−0.045
10%	−0.060	0.002	−0.171
2.5%	−0.338	${4.49\times 10}^{-4}$	−0.581
0.5%	−1.258	${8.37\times 10}^{-5}$	−1.652
Min	−4.005	${1.15\times 10}^{-8}$	−4.005
Mean	${1.04\times 10}^{-5}$	0.074	−0.076
Number	15,000	7609	7391

**Table 3:** Mean and median MPD values from the original and corrected profiles.

MPD Value (mm)			Baseline Length (mm)		
			1	5	10
Sample 1	Original	Mean	0.212	0.564	0.929
		Median	0.033	0.267	0.631
	Corrected	Mean	0.099	0.220	0.293
		Median	0.033	0.203	0.272
Sample 2	Original	Mean	0.236	0.698	1.123
		Median	0.051	0.565	1.024
	Corrected	Mean	0.099	0.223	0.294
		Median	0.033	0.204	0.272
Sample 3	Original	Mean	0.176	0.511	0.758
		Median	0.072	0.435	0.714
	Corrected	Mean	0.099	0.220	0.293
		Median	0.033	0.203	0.272
Sample 4	Original	Mean	0.115	0.315	0.475
		Median	0.054	0.242	0.420
	Corrected	Mean	0.099	0.220	0.294
		Median	0.033	0.203	0.273
